# Sociability and synapse subtype-specific defects in mice lacking SRPX2, a language-associated gene

**DOI:** 10.1371/journal.pone.0199399

**Published:** 2018-06-19

**Authors:** Breeanne M. Soteros, Qifei Cong, Christian R. Palmer, Gek-Ming Sia

**Affiliations:** 1 Department of Pharmacology, The University of Texas Health Science Center at San Antonio, San Antonio, TX, United States of America; 2 Department of Chemical and Biomolecular Engineering, Vanderbilt University, Nashville, TN, United States of America; Universita degli Studi dell'Insubria, ITALY

## Abstract

The FoxP2 transcription factor and its target genes have been implicated in developmental brain diseases with a prominent language component, such as developmental verbal dyspraxia and specific language impairment. How FoxP2 affects neural circuitry development remains poorly understood. The sushi domain protein SRPX2 is a target of FoxP2, and mutations in SRPX2 are associated with language defects in humans. We have previously shown that SRPX2 is a synaptogenic protein that increases excitatory synapse density. Here we provide the first characterization of mice lacking the SRPX2 gene, and show that these mice exhibit defects in both neural circuitry and communication and social behaviors. Specifically, we show that mice lacking SRPX2 show a specific reduction in excitatory VGlut2 synapses in the cerebral cortex, while VGlut1 and inhibitory synapses were largely unaffected. SRPX2 KO mice also exhibit an abnormal ultrasonic vocalization ontogenetic profile in neonatal pups, and reduced preference for social novelty. These data demonstrate a functional role for SRPX2 during brain development, and further implicate FoxP2 and its targets in regulating the development of vocalization and social circuits.

## Introduction

Speech and language are keystone abilities required for communication between members of a social group, and impaired language development is a prominent component of many neurodevelopmental brain disorders, including autism spectrum disorder (ASD) and schizophrenia. In the search for the genetic underpinnings of language behaviors, the foxhead-box protein P2 (FoxP2) transcription factor has emerged as a key player. Mutations in FoxP2 cause the only known monogenic language disorder in humans [1], and FoxP2 is involved in language and vocalization in multiple species [2–4]. Single nucleotide polymorphisms (SNPs) in FoxP2 are also associated with language impairments in autism [5,6] and schizophrenia [7,8]. FoxP2 exerts its effects through regulating the expression of a network of target genes [9,10], and is widely expressed in many neuronal populations [11–13]. Many FoxP2 target genes have been shown to be involved in various aspects of brain development [14–16], allowing FoxP2 to control the development of the multiple brain circuits that are expected to underlie complex behaviors such as sociability and language.

While a large number of FoxP2 targets have been identified, there has been fewer studies examining the effect of these genes on neural circuitry and animal behavior. The neurexin family membrane protein CNTNAP2 is a target of FoxP2 [17], and has been linked to language disorders [18] as well as autism [19–21]. The CNTNAP2 KO mouse shows abnormal neuronal migration, reduced numbers of interneurons in the striatum and hippocampus, stereotypic motor movements, reduced ultrasonic vocalizations, and impaired sociability behavior [22]. Another FoxP2 target, the Mef2C gene [23], is a transcription factor that has been linked to mental retardation and autism [24–26], and is known to negatively regulate excitatory synapse numbers [27]. The neuron-specific KO of Mef2c has been shown to reduce ultrasonic vocalizations in neonatal mice [23]. Hence, FoxP2 regulates a variety of processes involved in brain development, which in turn impacts a variety of behaviors.

The sushi domain protein SRPX2 is a target of FoxP2 [14,28]. SRPX2 encodes a secreted protein that regulates synapse formation and ultrasonic vocalization in mice [14], and mutations in SRPX2 in humans have been linked to language defects [29,30]. Here, we show that the SRPX2 knockout mouse shows a reduction in the VGlut2 subtype of excitatory synapses in the cortex, and displays an abnormal ultrasonic vocalization developmental profile and reduced preference for social novelty. This initial description of the SRPX2 KO mouse expands the list of developmental processes and brain circuits regulated by FoxP2 targets, and could provide a novel mouse model for investigating the role of these processes in language acquisition.

## Materials and methods

### Mice

Mice carrying an SRPX2 allele with exons 6 and 7 deleted were generated by CRISPR/Cas9 as described below, and maintained on the C57BL/6J background (Jackson Laboratories, Bar Harbor, Maine). Mice used for experiments were male littermates SRPX2^+/Y^ (WT) and SRPX2^-/Y^ (KO) derived from mating C57BL/6J male mice with a female mice heterozygous for the SRPX2 KO allele. We performed all experiments on male mice because SRPX2 is an X-linked gene and female WT and KO littermates cannot be generated from a single litter. Mice were group housed under a 12 h light/dark cycle and provided with ad libitum access to food and water. All procedures were approved by the UT Health Science Center Institutional Animal Care and Use Committees.

### Production and verification of SRPX2 KO allele with CRISPR/Cas9

**Fig 1:**
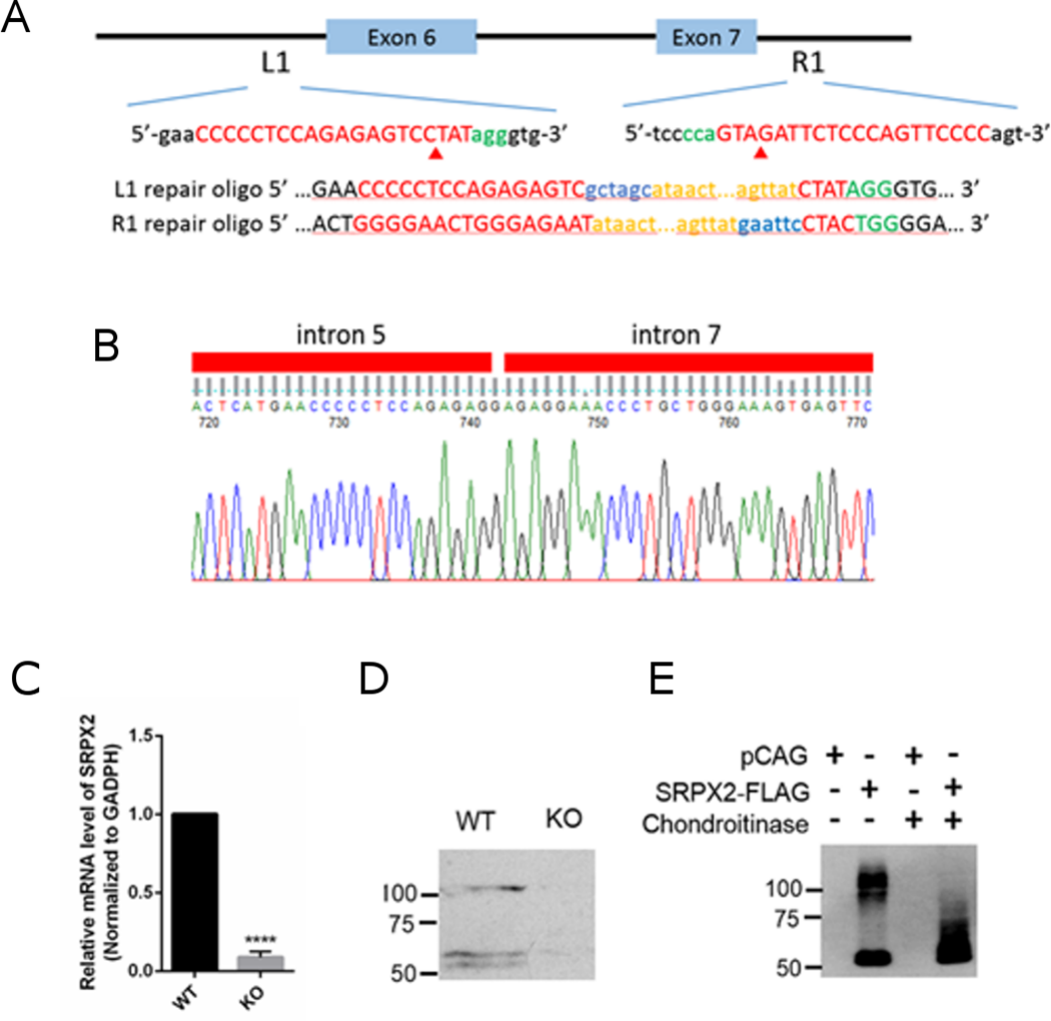
Generation of the SRPX2 KO mouse. (A) Schematic of Cas9/sgRNA/repair oligo targeting site in SRXP2 introns 5 and 7. sgRNA sequences are in red, PAM sequence in green, loxP sequence in gold, and restriction sites in blue. Arrow shows approximate site of Cas9-induced double stranded break. (B) Partial chromatograph of the genomic sequence of the SRXP2 KO mouse, showing deletion of exons 6 and 7, and a single G insertion at the junction. The deletion of exons 6 and 7 of the SRPX2 gene, and introduces a frameshift mutation which also truncates the gene shortly after exon 5, resulting in a null allele. (C) qRT-PCR of mRNA from the SRPX2 KO mouse brain which shows the absence of SRPX2 mRNA. (D) Western blot of brain lysates from P20 WT and SRPX2 KO mice for SRPX2 protein. (E) Chondroitinase ABC digestion of SRPX2 in conditioned medium from vector or SRPX2-FLAG transfected HEK cells.

We initially set out to engineer an SRPX2 conditional allele by inserting loxP sites into the introns flanking exons 6 and 7 of the *SRPX2* locus using CRISPR/Cas9 [31–33] (Fig 1A). This introduced a frameshift mutation and premature stop codons in the middle of the SRPX2 gene, which is expected to lead to nonsense-mediated decay of SRPX2 mRNA. We designed 2 sgRNAs targeting introns 5 and 7 of the *SRPX2* locus using the CRISPR Design website (http://crispr.mit.edu/). To ensure that any off-target editing events are not linked to the SRPX2 gene, we chose sgRNAs which do not have any off-target sites on the X chromosome. To facilitate the detection of the inserted loxP sequences, we also introduced restriction sites into the repair oligos. The sgRNAs, repair oligos, and Cas9 mRNA were co-injected into the 1 cell-stage zygotes of C57BL/6J mice, which were immediately transferred into pseudo-pregnant female mice. We obtained 29 founder mice from these injections, which were PCR-screened with primers surrounding the expected sites of editing. One of the founder mice was found to contain an SRPX2 allele with a large deletion that spans both editing sites, thereby deleting exons 6 and 7, but without a loxP insertion at either site (Fig 1B). This phenomenon has previously been reported [33], and is believed to be due to sgRNA introduced DNA breaks which are not repaired through the homology-directed repair (HDR) pathway, but instead through the non-homologous end joining (NHEJ) pathway. The resulting allele is equivalent to a conventional SRPX2 KO allele, and we therefore refer to mice derived from this founder as SRPX2 KO mice. We mated this founder mouse with C57BL/6J mice from Jackson Laboratories for 2 additional generations to dilute away any off-target editing sites, and then repeated the sequencing of the genomic locus with a different set of primers to verify that exons 6 and 7 had been deleted. We also verified that the SRPX2 mRNA is absent in the homozygous KO mouse using qRT-PCR (described below), and that the SRPX2 protein is absent with Western blotting (Fig 1D and 1E).

### Quantitative real-time reverse transcriptase polymerase chain reaction (qRT-PCR)

Brains were flash frozen in liquid nitrogen and stored in– 80°C until use. Total RNA was extracted using Ambion Trizol reagent (Fisher Scientific, Waltham, MA, USA) according to manufacturer’s protocol. mRNA was reverse transcribed using the SuperScript III First Strand Synthesis System (Invitrogen). qRT-PCR was performed in triplicate using a SYBR green PCR Master Mix (Applied Biosystems) on an ABI qPCR machine. The data were normalized to GAPDH and subsequently normalized to an experimental control group (ΔΔCT method). The primers used for qPCR were as follows: SRPX2 forward: GCTGCTTGACTCCCGTTGTG, SRPX2 reverse: GGCTCTGCCATTTTCTCACGA, GAPDH forward: AGGTCGGTGTGAACGGATTTG, GAPDH reverse: TGTAGACCATGTAGTTGAGGTCA.

### Golgi staining

Unfixed brains of 2 month old male mice were processed using the FD Rapid Golgi stain kit (FD Neurotechnologies, Columbia, MD). Halved brains underwent a 14 day impregnation period in kit solution A&B, followed by three 24-hour rinses in solution C with gentle shaking. Brains were flash frozen using isopentane and dry ice. Two-hundred micron sagittal sections were taken on a sliding microtome. Sections were mounted onto gelatin-coated slides and dried for 7 days. Slides were developed according to manufacturer’s instructions and coverslipped with Permount mounting medium (Electron Microscopy Sciences, Hatfield, PA). Neurons were imaged on a Zeiss Imager M.2 microscope and traced using Neurolucida software (MBF Bioscience, Williston, VT). For layer V neurons, dendritic spines were counted along 50–100 micron sections of the apical dendrite, starting at the base of the soma. For layer II/III neurons, dendritic spines were counted along the secondary basal dendrites. Dendritic spine density was calculated for three-to-five neurons per animal, three-to-four animals per group.

### Immunohistochemistry

Mice were perfused with ice-cold PBS and 4% paraformaldehyde (PFA). Brains were immediately removed and post-fixed in 4% paraformaldehyde for 48 hours, then cryoprotected in 30% sucrose in PBS for 48–72 hours. Free-floating 20 micron brain sections were briefly post-fixed in 4% PFA in PBS, washed 3x in PBS with 0.2% TX-100 (PBT), and blocked in 10% normal donkey serum in PBS. Sections were incubated with primary antibodies overnight at 4°C, washed 3x in PBT, and then incubated in secondary antibodies for 4 h at room temperature. Concentrations of antibodies are as follows: anti-VGLUT1 (1:5000, Millipore Sigma), anti-VGLUT2 (1:5000, Millipore Sigma), anti-PSD-95 (1:500, Neuromab), anti-VGAT (1:500, Synaptic Systems) anti-gephyrin (1:500, Synaptic Systems), anti-mouse IgG2a-A488 (1:500, ThermoFisher), anti-mouse IgG1a-A488 (1:500, ThermoFisher), anti-guinea pig A555 (1:500, ThermoFisher), DAPI (1:300, Invitrogen). Brainstain images were obtained with the Brainstain Imaging Kit™ (ThermoFisher), with staining was carried out in accordance with the manufacturer’s instructions.

Laser-scanning confocal fluorescence microscopy was performed using an Olympus FV-1000 confocal microscope. Images were captured using a Plan Apo 60x 1.3 NA oil-immersion objective with a Nikon camera. At zoom 1.5, 15 × 0.3 μm optical sections were acquired per slice. Image quantification was performed using ImageJ (NIH). Five optical slices from each stack were z-projected (summing intensities). Background subtraction was performed prior to thresholding. Images were binarized and multiplied for a pixel-to-pixel colocalization.

### Three chamber sociability and social novelty task

Two-month old male SRPX2-KO mice and WT littermates were subjected to the Crawley three-chamber sociability test, as previously described [34]. Mice were acclimated to the testing room for ~1.5 hours prior to the test. Subject mice and stranger mice were placed in different areas to avoid premature exposure. Stranger mice were familiarized with the pencil cups for two consecutive days prior to the experiment to reduce anxiety-like behavior. To begin the three-chamber assay, subject mice were habituated to the empty chamber for ten minutes. Next, wire pencil cups containing an age-matched male C57BL/6J stranger mouse or a novel object were randomly placed in either the left or right-side chambers. To begin the sociability phase, doorways to each side chamber were simultaneously opened, and subject mice were allowed to explore freely for ten minutes. Then, subject mice were briefly (~1–2 min) placed back in the center chamber, and the pencil cup with the inanimate object was removed and replaced with a novel mouse for the social novelty phase. Subject mice were then allowed to explore the familiar mouse, the middle chamber, or the novel mouse for 10 minutes. Chamber duration, sniff duration (nose within 1 cm of wire pencil cup), distance and velocity were recorded with an overhead camera and measured using Ethovision XT 11.5 automated tracking software (Noldus, Wageningen, Netherlands). After each experiment, the chambers and cups were thoroughly cleaned with the germicidal detergent LpH to remove any feces or urine. The chambers and cups were then sprayed with ethanol and allowed to aerate for 20 minutes to eliminate residual odors from the cleaning solutions or previous mice. The test room was dimly and evenly lit at all times.

### Infant pup isolation-induced ultrasonic vocalization (USV) task

Ultrasonic vocalizations were recorded from male pups at postnatal day 3, 6, and 9. Dams were separated from pups 15 minutes prior to testing. Pups were placed on a 30 mm petri dish inside a sound attenuation chamber (Med Associates Inc, Albans, VT, USA). Ultrasonic vocalizations were recorded using the Dodotronic Ultramic 384k ultrasonic microphone (Dodotronic, Italy), at a sampling frequency of 250 kHz. For identification purposes, toes were clipped after the recording session at P3. Only pups which underwent all three recording sessions were included in the analysis. Ultrasonic vocalizations were analyzed using the automated call detection feature in SYRINX (John Burt), using identical call picking parameters for both WT and KO.

### Statistics

For qRT-PCR, Golgi staining and immunohistochemistry, comparisons were made with unpaired, two-tailed Student’s T-test. Data were analyzed using Prism 7 software (Graphpad). Differences with p values < 0.05 were considered statistically significant. All data are plotted as mean ± SEM. For the three-chamber sociability assay, sociability and social novelty were analyzed using two-way repeated measures ANOVA, followed by Sidak’s multiple comparisons test. Student’s t-test was used to compare the social: non-social ratio [stranger/(middle/object)] across groups. For ultrasonic vocalization, the SRPX2-KO group did not pass the D’Agostino & Pearson normality test at p3, p6 or p9. Therefore, a two-tailed Mann Whitney U test was used to compare WT and SRPX2 KO groups at each day. The Holm-Sidak method was then used to adjust for multiple comparisons. Data for the studies are summarized in Supporting Information (S1 Table).

## Results

### Expression of SRPX2 in the mouse brain

**Fig 2:**
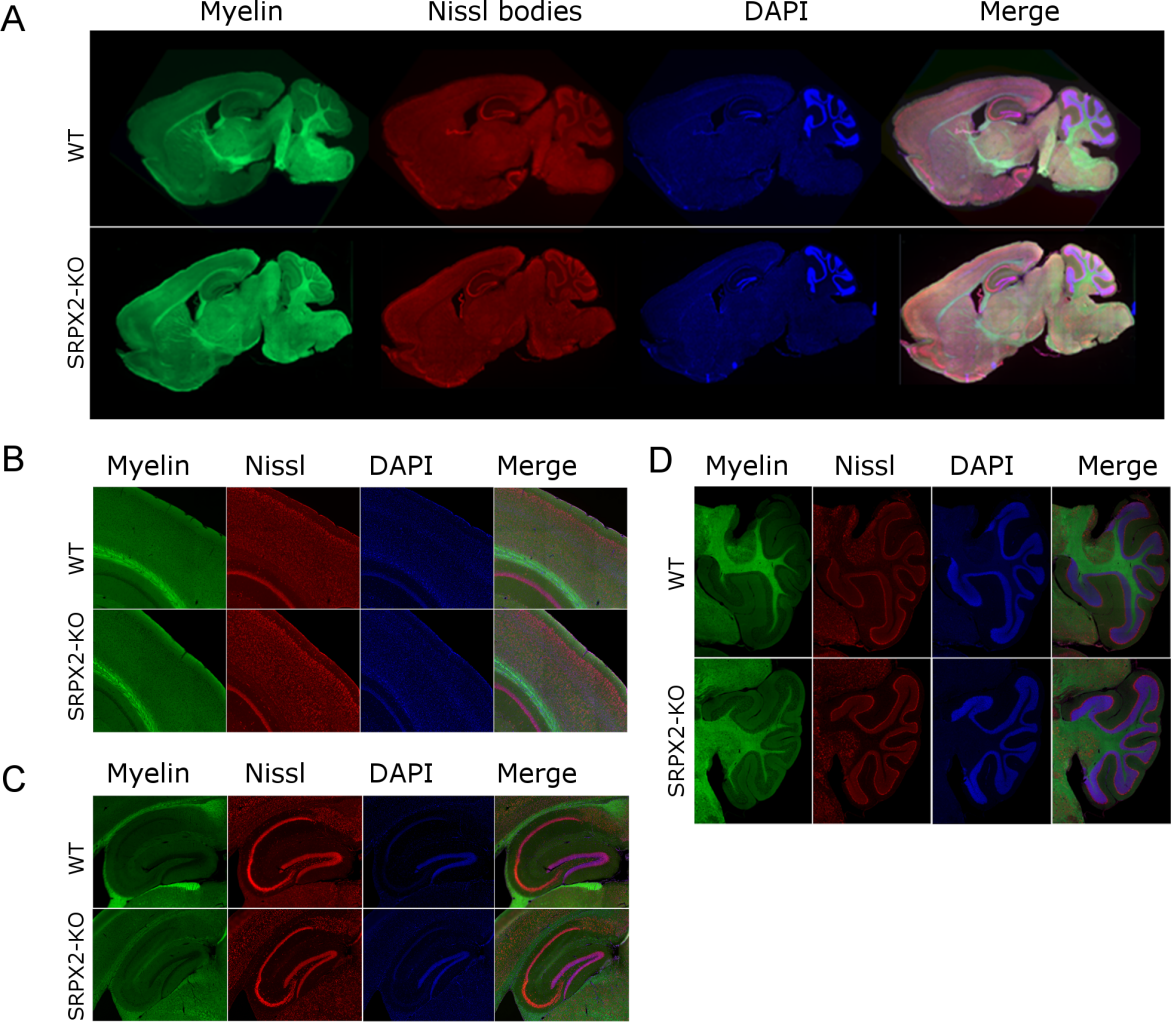
SRPX2 KO brains have no major defects in neuronal cell layers and axon tracts. Brainstain™ images of (A) whole brain, (B) cortex, (C) hippocampus, and (D) cerebellum for WT and SRPX2-KO mice.

In order to determine the consequences of SRPX2 gene deletion in mice, we employed CRISPR-Cas9 technology to create the SRPX2 KO mouse on the C57BL/6J background (Fig 1A and 1B), and verified absence of SRPX2 expression with genomic sequencing, RT-PCR and immunoblotting (Fig 1C and 1D). SRPX2 blots show a 50 kD protein band consistent with its predicted molecular weight, as well as a > 100 kD band, which is due to post-translational chondroitin modification (Fig 1E), as has been previously reported [35]. The SRPX2 KO mice produced offspring with the expected Mendelian ratio, showing that SRPX2 is not required for survival or fertility. Gross anatomy of the brain was normal with no defects in major neuronal cell layers and axon tracts in all regions of the brain (Fig 2).

### Loss of SRPX2 leads to decreased VGlut2 synapse density in layer IV of the somatosensory cortex

**Fig 3:**
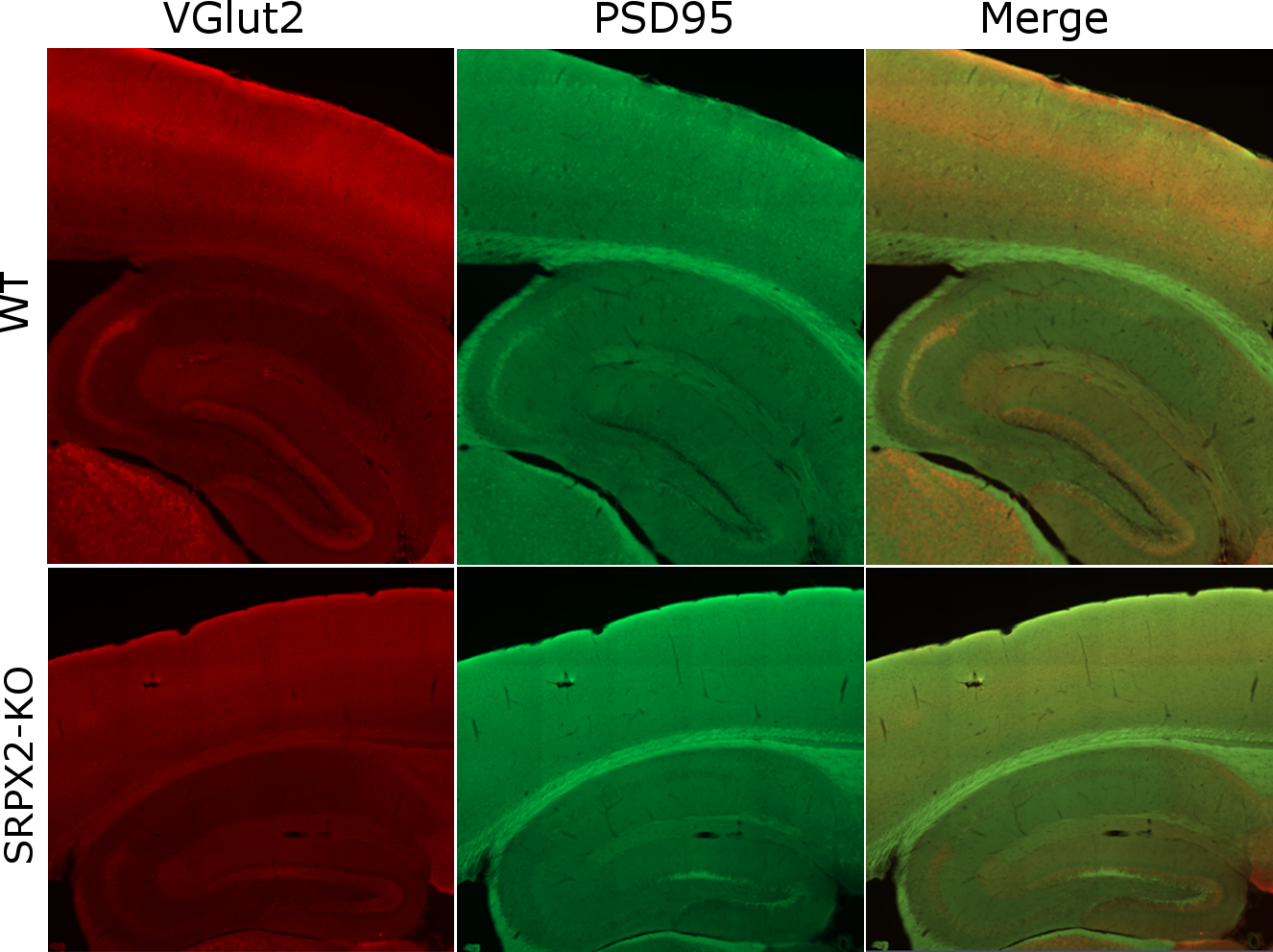
SRPX2 KO show a reduction of VGlut2 staining in the cortex. Low magnification images of cortex from WT and SRPX2 KO mice, stained for VGlut2 and PSD95, showing enriched VGlut2 staining in layer 4 of the SS cortex and CA2 of the hippocampus of WT mice, and reduction of VGlut2 staining intensity in SRPX2 KO mice.

**Fig 4:**
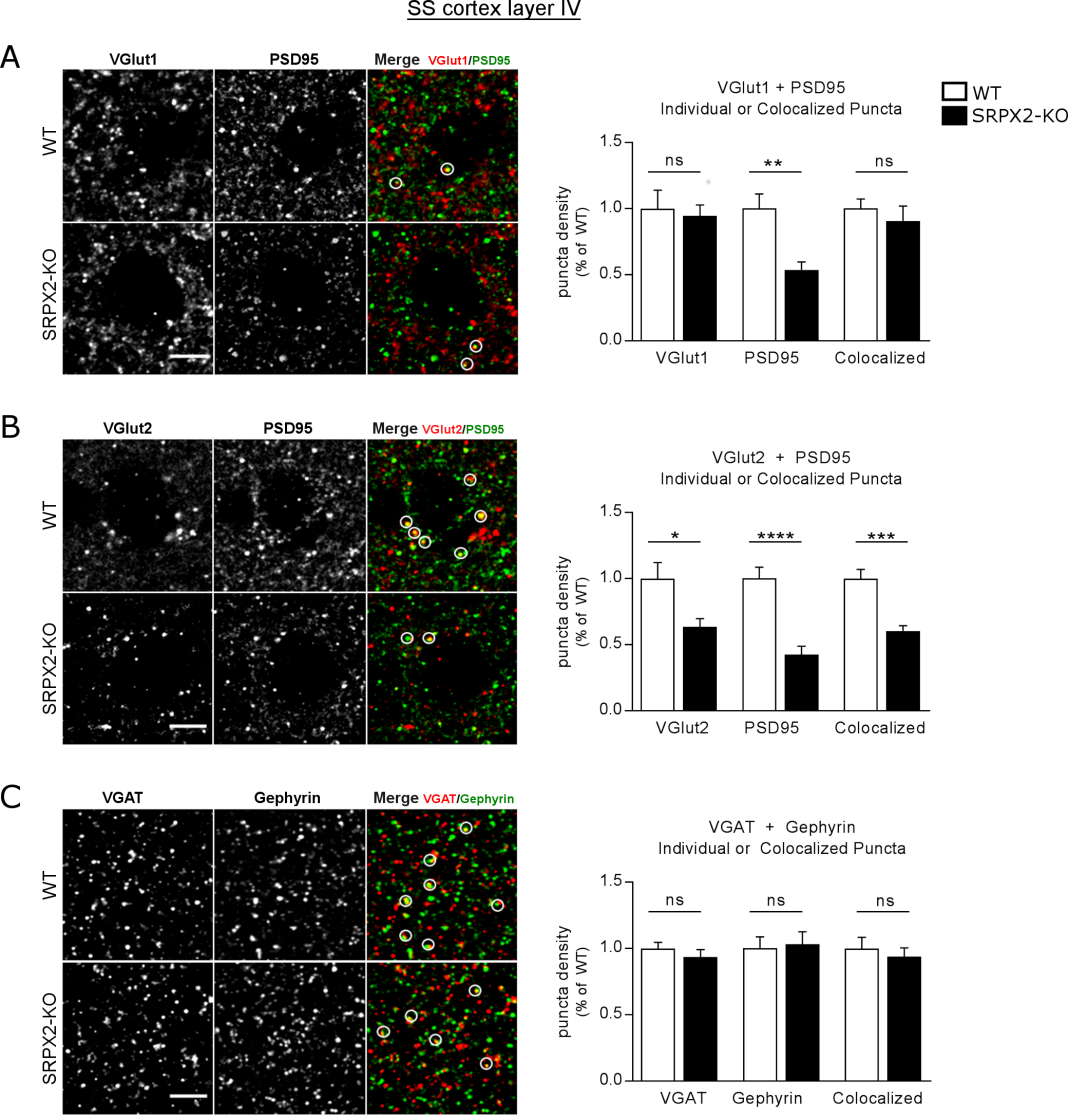
SRPX2 KO mice show a reduction of VGlut2 synapse density in layer IV of the SS cortex. Immunostaining of SS cortex layer IV for (A) excitatory presynaptic marker VGlut1 (red) and postsynaptic marker PSD95 (green), (B) excitatory presynaptic marker VGlut2 (red) and PSD95 (green), and (C) inhibitory presynaptic marker VGAT (red) and postsynaptic marker gephyrin (green). Synapses are composed of colocalized presynaptic and postsynaptic markers, indicated by circles. Scale bar 5 μm (A-C). Analyzed with Student’s t-test, two-tailed. n = 3–4 mice per group, 3–4 optical stacks per mouse. All data plotted as mean ± S.E.M. * p<0.05, ** p<0.01, *** p<0.001, **** p<0.0001.

**Fig 5:**
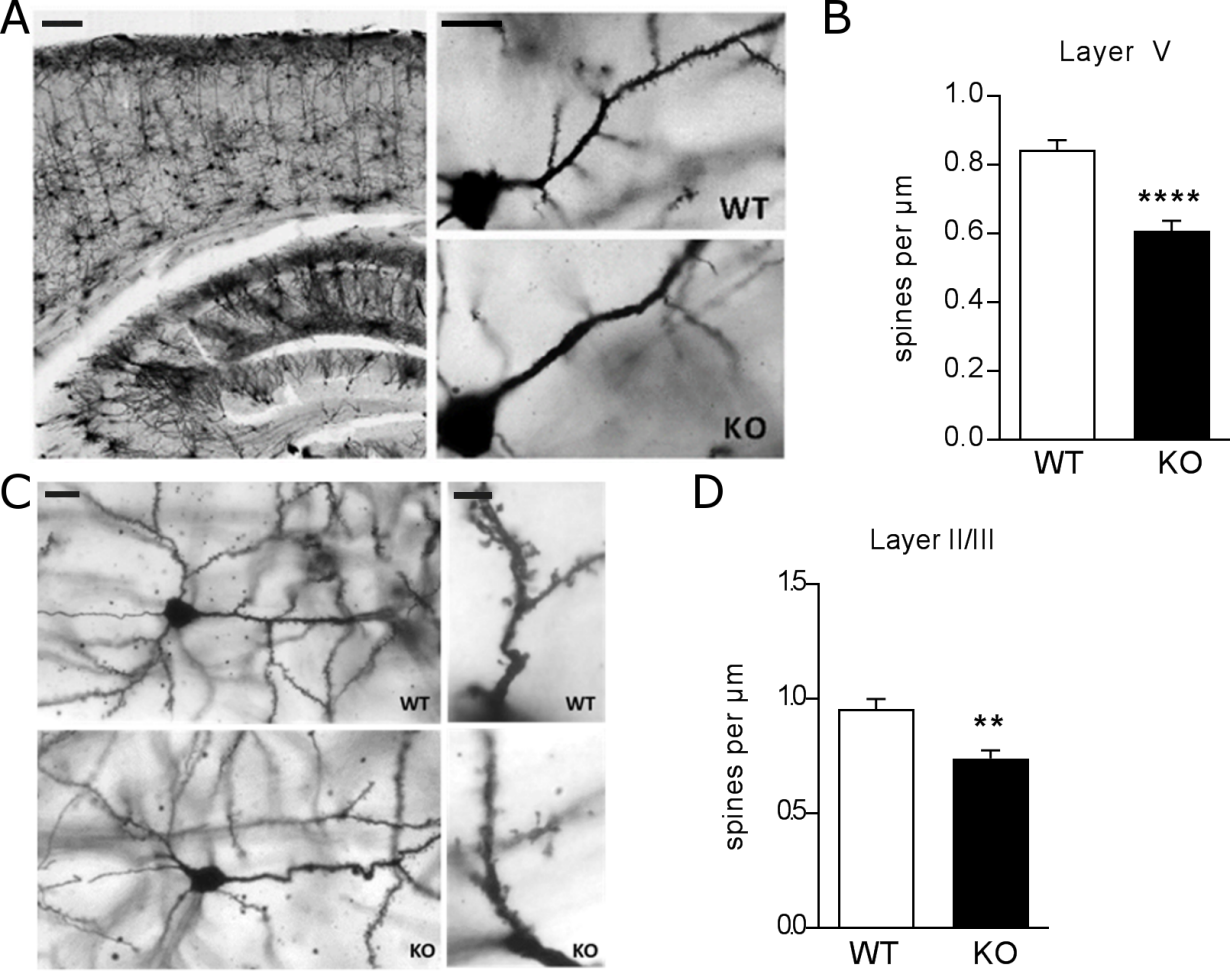
Reduction of spine density in apical dendrites of layer V and basal dendrites of layer III cortical neurons. (A) Representative images of Golgi-stained cortex of WT and SRPX2 KO mice. Scale bar 100 μm (left), 20 μm right. (B) Quantification of spine density on apical dendrite of layer V projection neurons in SS cortex. Analyzed with Student’s t-test, two tailed. *n* = 3–4 animals per group, 3–5 cells per animal, 10–12 cells total per group. (C) Representative images of layer II/III pyramidal neurons in WT and SRPX2 KO mice. Scale bar 20 μm (left), 5 μm right. (D) Quantification of spine density on basal dendrites of layer II/III pyramidal neurons. Student’s t-test, two tailed. *n* = 3–4 animals per group, 3–5 cells per animal, 10–12 cells total per group. All data plotted as mean ± S.E.M. ** p<0.01, **** p<0.0001.

We have previously shown that SRPX2 is a synaptogenic protein that increases excitatory synapse density when applied to cultured cortical neurons, suggesting that SRPX2 may play a role in synapse formation during development of the cortex [14]. ISH data show that SRPX2 is broadly expressed in diverse populations of neurons, including in the cortex [29,36]. To examine the effects of SRPX2 deletion on cortical synapse formation, we performed immunohistochemistry in the mouse cortex for the excitatory synapse markers VGlut1, VGlut2 and PSD95, as well as with the inhibitory synapse markers VGAT and gephryin as controls. We observed a marked reduction in VGlut2 staining that is visible even under low magnification (Fig 3), especially over areas in the cortex which are enriched for VGlut2, including layer IV of the somatosensory (SS) cortex and the CA2 region of the hippocampus [37–39]. We performed high resolution confocal microscopy to visualize excitatory synapses, indicated by colocalized VGlut1/VGlut2 and PSD95 puncta, and inhibitory synapses, indicated by colocalized VGAT and gephyrin puncta. We observed a prominent reduction of VGlut2 synapses in layer IV of the SS cortex (Fig 4B), without any changes in the densities of VGlut1 and VGAT synapses (Fig 4A and 4C), indicating that the effect of SRPX2 is specific to the VGlut2 subpopulation of synapses in this brain region. Layer IV of the SS cortex is heavily innervated by axons from thalamic neurons which express VGlut2 but not VGlut1 [40,41], while most cortical neurons express VGlut1 but not VGlut2, suggesting that SRPX2 may regulate the density of thalamocortical synapses in the SS cortex. To verify this with an antibody-independent method, we turned to Golgi-Cox staining, which allows for quantification of dendritic spine densities, an anatomical correlate of excitatory synapses. The most direct approach would be to examine dendritic spine density of layer IV neurons which are known to be heavily innervated by thalamic inputs. However, in the SS cortex, layer IV neurons are arranged in cell arrays termed ‘barrels’, and the dendritic spine densities of these neurons vary substantially based on their location within the barrel. While barrels can be clearly seen under DAPI/Nissl staining, they are difficult to discern under Golgi staining when < 1% of neurons are labelled. We have found that when layer IV neuronal spine densities are directly quantitated, there is substantial variation even within the same brain section. We therefore decided to focus on dendrites from other cell layers that traverse layer IV, specifically, apical dendrites of layer V pyramidal neurons and basal dendrites of layer II/III neurons. We found that these dendritic segments show a consistent spine density within layer IV irrespective of their positions in the barrel. Classical anatomical methods have shown that thalamic inputs synapse on basal dendrites of layer II/III pyramidal cells, as well as the apical dendrites of layer V pyramidal neurons. This connectivity has been verified using more modern techniques, such as optogenetic mapping of thalamic VPM projections into the neocortex [42], as well as detailed EM reconstruction of thalamocortical circuits [43]. Both of these dendritic segments show a reduction in spine density in the SRPX2 KO mouse (Fig 5), which is consistent with a decrease in thalamocortical inputs into the SS cortex.

### Loss of SRPX2 leads to decreased VGlut1, VGlut2 and VGAT synapse densities in the CA2 region of the hippocampus

**Fig 6:**
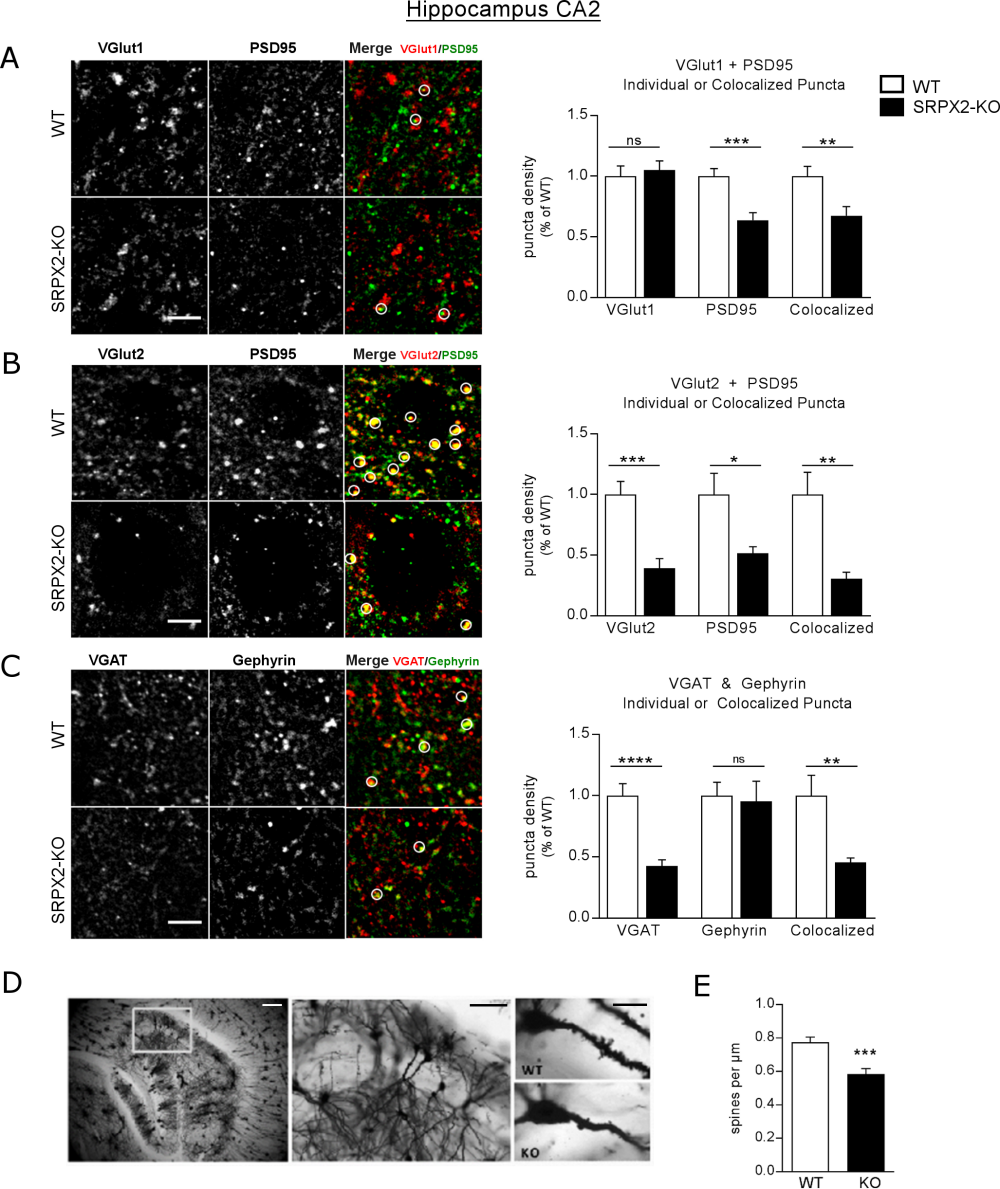
Reduction of multiple synaptic markers in hippocampal CA2 of SRPX2 KO mice. Immunostaining of CA2 for (A) excitatory presynaptic marker VGlut1 (red) and postsynaptic marker PSD95 (green), (B) excitatory presynaptic marker VGlut2 (red) and PSD95 (green), and (C) inhibitory presynaptic marker VGAT (red) and postsynaptic marker gephyrin (green). Synapses are composed of colocalized presynaptic and postsynaptic markers, indicated by circles. Scale bar 5 μm (A-C). Analyzed with two-tailed Student’s t-test, *n =* 3–4 subjects per group. (D) Representative images of Golgi-stained CA2 of WT and SRPX2-KO mice. Scale bar 100 μm (left, middle), 20 μm (right). (E) Quantification of spine density on apical dendrites of CA2 pyramidal neurons. Student’s t-test, two-tailed. *n* = 3–5 subjects per group, 3–5 cells per subject. 10–12 cells total per group. All data plotted as mean ± S.E.M. * p<0.05, ** p<0.01, *** p<0.001, **** p<0.0001.

To determine if the defect in VGlut2 synapses is generalizable to other brain regions, we also examined synapse densities in the CA2 region of the hippocampus. In CA2, VGlut2 is derived from mossy fiber innervation from the dentate gyrus (DG) [44,45]. Unusually among axon projections, the mossy fibers of the DG contain multiple vesicular transporters, namely VGlut1, VGlut2 and VGAT [45,46]. Optogenetic mapping of the DG-CA2 pathway has also shown that DG mossy fibers innervate the apical dendrites of CA2 neurons [47]. In SRPX2 KO mice, we observed a decrease in the density of VGlut2 and VGAT puncta, and a decrease in the colocalization of VGlut1/VGlut2/VGAT puncta with their respective postsynaptic markers in the CA2 region of the hippocampus (Fig 6A, 6B and 6C). We also observed a drop in the spine density on the apical dendrites of CA2 pyramidal neurons by Golgi staining (Fig 6D and 6E). Together, this data suggests that mossy fiber innervation of CA2 is decreased in SRPX2 KO mice.

### SRPX2 KO mice show an abnormal USV ontogenetic profile

**Fig 7:**
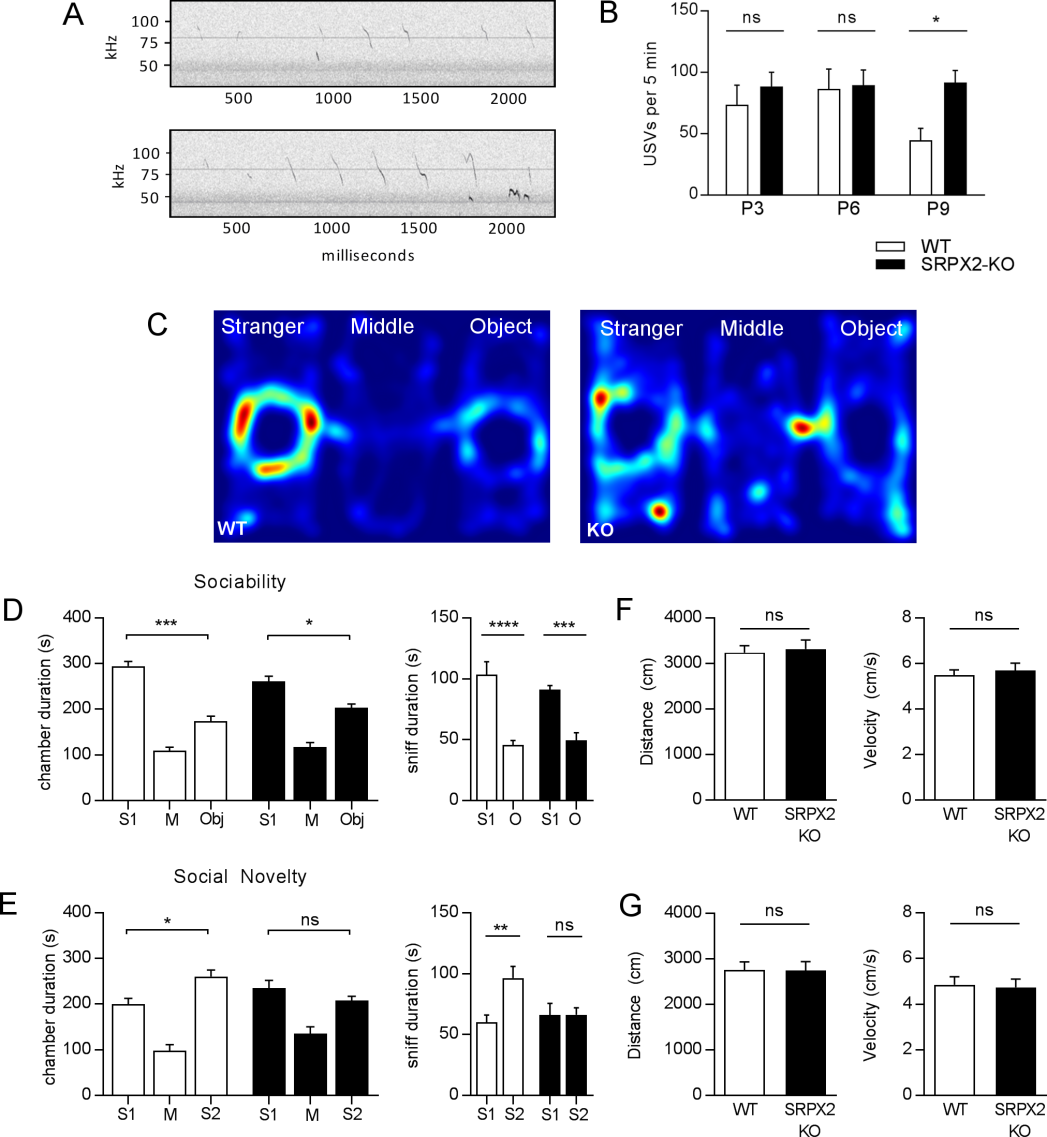
Deficits in vocalization and social behaviors in SRPX2 KO mice. (A) Representative spectrograms of isolation-induced ultrasonic vocalizations (USVs) for P9 WT and SRPX2 KO pups. (B) Quantification of USVs emitted over 5-minute periods. Pups tested at postnatal day 3, 6 and 9. Vocalizations of SRPX2-KO pups remain elevated at P9 compared to WT. Analyzed by Mann-Whitney U, p = 0.0203. n = 17–48 per group. (C) Representative heat maps from the three-chamber sociability and social novelty task. (D) Left panel, Time spent in chamber containing stranger mouse 1 (S1), middle empty chamber (M), and chamber containing inanimate object (Obj). Analyzed by two-way repeated measures ANOVA, F(2,34) = 3.743, p = 0.0340. Right panel, time spent sniffing stranger and object. Analyzed by one-way ANOVA, F(3, 34) = 20.03, p<0.0001). (E) Left panel, time spent in the chamber containing the original stranger mouse 1 (S1), the middle empty chamber (M), and the chamber containing the novel stranger mouse (S2). Two-way repeated measures ANOVA, F(2, 34) = 4.834, p = 0.0142. Right panel, time spent sniffing familiar mouse (S1) and novel stranger mouse (S2). One-way ANOVA, F(3,34) = 4.078, p = 0.0141. (F) and (G) Distance and velocity of WT and SRPX2 KO mice during each phase of the 3-chamber sociability task. Student’s t-test, two-tailed. n = 9–10 per genotype (D-G). All data plotted as mean ± S.E.M. One and two-way ANOVAs followed by Sidak’s multiple comparisons test. * p<0.05, ** p<0.01, *** p<0.001 **** p<0.0001.

In order to determine if SRPX2 deletion leads to any behavioral phenotypes, we first tested the SRPX2 KO mice in the infant pup isolation-induced ultrasonic vocalization (USV) behavioral task. Neonatal mouse pups emit ultrasonic vocalizations (USVs) when they are removed from their nest, generally in the 30–90 kHz range [48]. USV production is a reflexive behavior, and is not learnt and does not depend on auditory feedback. Neonatal USVs elicit a search-and-retrieve behavior in the mother, and the USVs are potentiated by olfactory cues signaling the presence of the mother, and suppressed by cues signaling the presence of a stranger male. USVs are also modulated by room temperature, as well as the affective state of the pup. Considerable differences in vocalization rate have been found in different strains of inbred mice, with C57BL/6J mice showing generally less vocalization [49]. Neonatal USVs also follow a characteristic ontogenetic profile, with calls increasing from birth and reaching a peak at postnatal day 4–6 (P4-6), and then declining to zero at P14-15 [48]. This task has been widely used previously to examine the development of vocalization behavior in mice in the context of a variety of social and arousal disorders, including in mice bearing mutations in the FoxP2 gene [50–55]. In the SRPX2 KO infant pups, we observed an abnormal USV ontogenetic profile, with increased vocalizations at P9 (Fig 7A and 7B).

### SRPX2 KO mice show reduced preference for social novelty

Because the CA2 region of the hippocampus has previously been implicated in social memory [56], and the SRPX2 KO mice has severe synaptic defects in this brain region (Fig 5), we also tested adult SRPX2 KO mice in the three-chamber sociability and social novelty task. This task is commonly used to screen mouse models of autism [57], and consists of two phases. The first phase allows the subject mouse to choose between the chamber containing an object and another containing a live mouse, and the time spent in the chamber with the live mouse is a measure of the sociability of the subject mouse. In the second phase, the inanimate doll is replaced with a novel live mouse, and the time the subject mouse spends in the chamber with the novel mouse is a measure of its preference for social novelty and social memory. SRPX2 deletion has minimal effects on the sociability of mice, but profoundly reduced their preference for social novelty (Fig 7C, 7D and 7E). Locomotion behavior was unaffected by SRPX2 deletion during the task (Fig 7F and 7G). The reduction in preference for social novelty is also apparent when we measured the time spent sniffing the novel vs the familiar mouse, which is a more sensitive measure in this task (Fig 7D and 7E). In summary, the absence of SRPX2 causes defects in both vocalization and social behavior in mice.

## Discussion

There is strong interest in FoxP2 and its target genes as molecular windows into the biological mechanisms that underlie language acquisition. Here, we show that mice that lack SRPX2, a FoxP2 target gene, exhibit reduced VGlut2 synapses in the cortex, and show behavioral phenotypes that resemble symptoms shown by ASD patients. SRPX2 KO mice have intact vocalization and locomotor behavior (Fig 5), but show an abnormal USV developmental profile. In addition, SRPX2 KO mice show reduced preference for social novelty. These studies suggest that language and social cognition abilities are closely linked at the molecular level [58,59].

### SRPX2 and brain regions underlying language development in mice

Research on FoxP2 and its targets have largely concentrated on neural circuitry of the basal ganglia as the neural basis of language acquisition. The basal ganglia play a well-established role in motor control, including speech production, and is therefore a reasonable substrate for language acquisition. In mice bearing humanized FoxP2 alleles, medium spiny neurons in the basal ganglia show longer dendritic lengths and increased synaptic plasticity [60]. KOs of the FoxP2 targets CNTNAP2 and Mef2C also show changes in basal ganglia circuitry [22,23]. Furthermore, research in songbirds have revealed that Area X, a songbird basal ganglia nucleus, play an exclusive and necessary role in song learning [4]. However, in humans, lesions of the Broca’s and Wernicke’s cortical areas are associated with aphasias, suggesting that cortical areas also play a role in speech production and comprehension. More recent studies in songbirds have also revealed a role for both sensory and motor cortices in song learning [61,62]. In this study, we found that the most prominent synaptic defect in SRPX2 KO mice is a striking reduction in the number of VGlut2 synapses in the cerebral cortex, with other synapse types being largely unperturbed. This is a surprising finding since SRPX2 is expressed in diverse populations of neurons, and it is unclear why only a subpopulation of synapses are affected. Since VGlut2 synapses in the cortex are associated with thalamic sensory inputs, our data suggests that SRPX2 regulates the formation of sensory inputs into cortical areas in mice. Another FoxP2 target, the MET receptor tyrosine kinase, is also involved in the growth and survival of sensory axons [63]. The selective vulnerability of VGlut2 synapses to SRPX2 deletion suggests that FoxP2 repression of its target genes may regulate the timing of sensory synapse formation in the sensory cortices, and thereby affect sensorimotor integration which is an essential step in the development of vocalization [61,62].

### SRPX2 and autism-related behaviors

A major ethological purpose of communication is to facilitate social behavior, and co-evolution of communicative abilities and social cognition has been previously postulated [58,59]. Indeed, while FoxP2 itself is not associated with autism [64,65], many of its targets are strong candidates as autism susceptibility genes, including CNTNAP2 [19,20] and MET [66,67]. In this study, we show that the SRPX2 KO causes defects in both vocalization development and social cognition. Both of these defects have also previously been seen in other mouse models of developmental psychiatric disorders. For example, an abnormal USV developmental profile with an elevated call rate at P9-10 has been seen in the Ts65Dn mouse model of Down’s syndrome [68] and the Purkinje cell Tsc1 KO mouse model of autism [69]. Reduced preference for social novelty has also been seen in mouse models associated with autism, including KOs of NLGN4 [70] and FGF17 [71] genes. In summary, our findings show that SRPX2 deletion disrupts multiple populations of VGlut2 synapses that are required for the development of circuits that modulate language and social behaviors.

### SRPX2 mechanism of action and discrepancy between IUE and KO USV phenotypes

Two previous studies have utilized in utero electroporation (IUE) shRNA knockdown of SRPX2 in cortical neurons to examine its role in brain development. IUE knockdown of SRPX2 in rats resulted in a neuronal migration defect [72], whereas IUE knockdown of SRPX2 in layer V/VI cortical neurons in mice resulted in a decrease in spine density in the apical dendrites and a reduction in the frequency of USVs in the isolation induced infant pup USV task [14]. In this study, we found that global deletion of the SRPX2 gene in mice causes a similar defect at the cellular level, with a reduction of spine density in apical dendrites of layer V neurons, but a different phenotype at the organism level, with an altered USV developmental profile rather than an outright reduction in vocalization. This suggests that SRPX2 may act through a competitive mechanism, where circuitry function is more dependent on intercellular differences in relative levels of SRPX2 rather than absolute levels of SRPX2. Such competitive mechanisms are widespread in the early development and refinement of sensory circuits [73,74]. Under this scenario, loss of a gene in a subset of neurons may lead to a more severe phenotype compared to a loss of the gene in all neurons. This behavior has been described in the synaptogenic protein neuroligin-1 (NL1), where synapse numbers are not sensitive to absolute NL1 levels but are instead dependent on transcellular differences in the relative amounts of NL1 [75]. Validation of this explanation would require additional data from cell-specific knockouts of SRPX2. SRPX2 is a secreted sushi domain protein, and is therefore expected to act through receptors in the extracellular compartment. In the innate immune system, sushi domain proteins are known to regulate the complement cascade [76]. More recently, the classical complement cascade has been found to regulate activity-dependent synapse elimination in the visual system [77]. We speculate that SRPX2 may function in brain development by inhibiting the complement pathway, thereby affecting competitive complement-dependent synapse elimination processes.

### Sushi domain proteins and brain development

SRPX2 is a member of the sushi domain protein family, which historically has been primarily studied as regulators of the complement cascade in the innate immune system [76]. More recently, sushi domain proteins have also been shown to play roles in brain development. In invertebrates, sushi domain proteins have also been shown to cluster acetylcholine receptors at the neuro-muscular junction of C. elegans [78], and in the Drosophila brain [79,80]. However, the functions of sushi domain proteins in the mammalian brain are largely unknown, although the sushi domains on the GABA_B_ receptor are known to function as axon-targeting signals [81]. Based on the HUGO Gene Nomenclature Committee annotation of the human genome, there are 57 sushi domain-containing genes in the human genome. Several of these genes have been associated with neurological diseases such as epilepsy and schizophrenia [82–85], including SRPX2 [29,30]. About half of all sushi domain genes in the human genome are expressed in the brain, according to the Allen Brain Atlas, and the function of most of these proteins are unknown. This study is one of the first descriptions of the role of a sushi domain protein in mammalian brain development, and sheds light on the functions of the numerous other orphan sushi domain proteins expressed in the brain, as well as suggests additional brain regions to examine in future studies examining FoxP2's effect on brain development.

## Supporting information

S1 Table Supplementary table with data for experiments shown in Figs 4, 5, 6 and 7. (XLSX)
